# Impulsivity and Response Inhibition Related Brain Networks in Adolescents With Internet Gaming Disorder: A Preliminary Study Utilizing Resting-State fMRI

**DOI:** 10.3389/fpsyt.2020.618319

**Published:** 2021-01-15

**Authors:** Jieyu Chen, Xinyi Li, Qun Zhang, Yu Zhou, Rongpin Wang, Chong Tian, Hui Xiang

**Affiliations:** ^1^Department of Medical Psychology, College of Medical Humanities, Guizhou Medical University, Guiyang, China; ^2^Binzhou Medical University, Binzhou, China; ^3^Department of Psychology, Guizhou Normal University, Guiyang, China; ^4^Department of Psychiatry, Guizhou Provincial People's Hospital, Guiyang, China; ^5^Department of Radiology, Guizhou Provincial People's Hospital, Guiyang, China

**Keywords:** internet gaming disorder (IGD), prefrontal-striatal circuits, functional magnetic resonance imaging (fMRI), voxel-mirrored homotopic connectivity (VMHC), effective connectivity

## Abstract

**Background and Aims:** Internet gaming disorder (IGD), as a relapse disease, has become a common mental health problem among Asian teenagers. Functional connections in the prefrontal lobo-striatum affect changes in impulsivity and inhibition. Therefore, exploration of the directional connections of the relevant brain regions in the prefrontal-striatal circuit and the synchronization level of the two hemispheres will help us to further understand the neural mechanism of IGD, which can provide guidance for the development of prevention and intervention strategies.

**Methods:** Twenty-two adolescents with IGD, recruited through various channels, composed the IGD group. Twenty-six subjects, matching age, gender, and education level, were included in a recreational internet game users (RGUs) control group. Impulsivity and response inhibition were tested via general questionnaire, the Internet Addiction Test (IAT), the Barratt impulsivity scale-11 (BIS-11), and a Stroop color-word task. A Granger causality analysis (GCA) was used to calculate the directional connection between the prefrontal and striatum with the dorsolateral prefrontal cortex (DLPFC) as a region of interest (ROI). We chose voxel-mirrored homotopic connectivity (VMHC) to determine brain hemisphere functional connectivity in the prefrontal-striatal circuits.

**Results:** We found significant differences in impulsivity between the IGD group and RGU group, with members of the IGD group exhibiting higher impulsivity. Additionally, the response inhibition of adolescents with IGD in the Stroop color-word task was impaired. There was a significant difference in the directed connection of the left DLPFC and dorsal striatum between the IGD group and the RGU group.

**Conclusions:** This study confirmed the role of prefrontal-striatal circuits in the neural mechanism of IGD in adolescents. In the IGD group, bilateral cerebral medial orbitofrontal cortex (mOFC) synchronization was significantly reduced, which indicated that mOFC signal transmission in both hemispheres of the brain might be affected by impulse behavior and impaired response inhibition.

## Introduction

The “42nd China Internet Development Statistics Report,” published by the China Internet Network Information Center, showed that the number of Internet users in China had reached 802 million, with an internet penetration rate of 57.7% by June 2018. The diversification of online games and game content was more obvious. With the popularity of the Internet, internet gaming disorder (IGD) (1) has had a considerable impact on public health.

IGD refers to the continuous and repeated use of the Internet to participate in games with others, resulting in clinically significant damage or pain. The Diagnostic and Statistical Manual of Mental Disorders (fifth edition) has classified IGD as a disease that requires further study. The 11th edition of the International Classification of Diseases (ICD-11) was released in June 2018, with game addiction being added to the list of behavioral addiction disorders. Adolescents and children are the main target population of online games. A systematic review of more than 50 studies worldwide (2) suggested that IGD is more common in adolescents.

Not everyone who comes into daily contact with online games develops IGD (3). Palaus et al. (4) collected the results of 116 scientific studies and found that online games could optimize attention resources, improving working memory and visual-spatial skills. This suggests that online games do not necessarily result in negative consequences and that IGD susceptibility varies among individuals (5). Therefore, this research recruited recreational online game users (RGUs) as a control group. While RGUs play online games for entertainment, they are not addicted and do not meet the diagnostic criteria of the Diagnostic and Statistical Manual of Mental Disorders (DSM−5) for IGD (6). A comparison between RGUs and individuals with IGD is beneficial to further understand online game addiction and the cause of addiction.

The abnormal psychological processes and behavior observed in individuals with IGD appear related to high levels of impulsivity and dysfunctional response inhibition (7). Some researchers define impulsiveness as a tendency to act too early and without careful consideration (8). Response inhibition refers to the ability to suppress responses that do not meet current demands or inappropriate behaviors. Impaired response inhibition is considered to be an important cause of impulsiveness (9). Some studies (10, 11) have observed abnormal activation of brain areas associated with impulsivity and inhibitory function in individuals with IGD. However, differences in brain functionality, based on changes in neural circuits, were not enough to explain abnormalities in external behavior.

Within the functional connectivity of anterior cingulate cortexes during the resting state, in contrast to the controls, the individuals with IGD showed increased connectivity with posterior cingulate, medium cingulate, midbrain, nucleus accumbens and supplementary motor area, but reduced connectivity with prefrontal cortex, temporal lobe, and occipital lobe. The IGD group had lower gray matter volume in the left orbitofrontal cortex, left medial prefrontal cortex, bilateral insula, left posterior cingulate cortex, and left supplementary motor area (12). These changes in brain structure might result in uncontrolled behavior and online gambling urges.

Some studies (13, 14) have reported dysfunction in frontal-striatal circuits in individuals with addiction. The prefrontal cortex and corpus striatum are regulated via dopaminergic connections. Video games might cause a rapid increase in striatal dopamine (15), making prefrontal-striatal circuits more likely to be activated. Response inhibition is primarily related to brain areas in the prefrontal cortex. Specifically, the hyper-direct and indirect pathways in the frontal-striatal circuits might be responsible for the inhibition of dominant responses. Impulsivity is also affected by prefrontal-striatal circuits, which play an important role in activating and inhibiting impulsive behavior (16).

One study (17) selected the dorsolateral prefrontal cortex (DLPFC) and ventral striatum as regions of interest to investigate the role of the prefrontal-striatal circuits in substance addiction. A previous study (18) had reported differences in rest-state functional connectivity (RSFC) of the prefrontal-striatal circuits in substance users. This is consistent with the conclusion of IGD studies (19). Further studies (20) have identified an association between RSFC and cognitive control deficits in patients with IGD. While abnormal cognitive control in patients with IGD has been shown to be associated with changes in the DLPFC, the current study focused only on the strength of the functional connections between the prefrontal cortex and the striatum. It is not clear whether the striatal system demonstrates “high impulsiveness,” the prefrontal system demonstrates “low control,” or a combination of dysfunction in these two aspects is responsible for IGD behavior. Therefore, this study intended to explore the direct brain network between the prefrontal cortex and striatum in adolescents with IGD, in an effort to determine causal effects and direction between nodes (21). According to previous studies (7, 8, 22, 23), the DLPFC, which plays an important role in inhibition control, was selected as a region of interest (ROI). Further, a Granger causality analysis (GCA) was used to determine the connection efficiency between DLPFC and striatal regions. Simultaneously, using voxel-mirrored homotopic connectivity (VMHC), the current study detected brain hemisphere differences in the striatum and prefrontal cortex synchronicity of the two groups.

This study hypothesized that, compared with RGUs, adolescents with IGD would exhibit significant differences in behavioral performance on an impulsivity index and Stroop color-word task, and that impulsivity would be correlated with Internet Addiction Test (IAT) scores (24). We also hypothesized that there would be significant differences between the two groups on connection efficacy of the prefrontal-striatal circuits, and in the VMHC values of related brain regions. The purpose of this study was to explore the prefrontal-striatal circuits and resting state functional connectivity, which are related to impulsiveness and response inhibition, in adolescents with IGD, so as to provide neurological explanations for online gaming behaviors in this population, and to provide new imaging targets for the clinical diagnosis and intervention of IGD.

## Methods

### Participant Selection

We conducted IAT questionnaire screening in middle schools in Guizhou Province. And we also screened through the Internet IAT questionnaire. A professional psychiatrist diagnosed adolescents with IAT>50 in Guizhou Provincial People's Hospital. Ninety-one adolescents (12–18 years of age), regardless of gender, were invited. Sixty-one adolescents declined the invitation, and 30 adolescents accepted the invitation. The criteria for being included in the IGD group were (1) an IAT score ≥ 50 (4, 5); (2) the main purpose of using the Internet was for playing online games; (3) an average daily online game time of 4–6+ h for more than 2 years (4, 5); (4) the symptoms met the diagnostic criteria for IGD in the DSM-5; (5) being right-handed. The criteria for being included in the RGU group were (1) IAT score <50 points; (2) an average daily online game time of 2–4 h for more than a year (4, 5); (3) being right-handed. The exclusion criteria were as follows: (1) psychoactive substance dependence, including coffee and cigarettes, schizophrenia, or other organic brain diseases; (2) severe cognitive dysfunction, such as head trauma history, cerebrovascular disease, or epilepsy; (3) other mental disorders; (4) taking medicine 1 week before the examination.

Strict quality control was carried out in this study. We have uniformly trained the study staff and had consistent test for all questionaires. Middle and high school students who met the screening standards in the psychiatric department of our hospital were selected from October 2016 to September 2018. A total of 30 eligible subjects were selected to be included in the IGD group. Matching the IGD group in terms of gender, age, and years of education, we recruited junior and senior high school students through online advertisement. A total of 30 subjects that met these criteria were selected to be included in the RGU group.

This study was approved by the ethics committee of the Guizhou Provincial People's Hospital. All of the subjects and their statutory guardians in the study were informed of the process and purpose of the study before the study started and provided written informed consent.

### Instruments

#### IAT (24)

The IAT is composed of 20 items rated on a 5-point scale, (1= *very rarely*, 5 = *very frequently*). All of the item scores are summed to obtain an IAT total score. A total of 50 points and below indicates no problem with internet addiction (IA); 50–79 indicates mild IA; 80–100 indicates severe IA.

#### Self-Designed Online Game Questionnaire

The questionnaire contained four questions:(1) How long have you been connected to the Internet? (2) What is the main purpose of your internet use? (3) Which of the following do you spend the most time on? (A. Game, B. Other), and (4) How long have you played games each day over the past year? If the answers to questions 2 and 3 were “game,” the time spent was 4–6+ h/day, and the subject's IAT score > 50 points, the individual was classified as exhibiting IGD.

#### Barratt Impulse Scale, 11th Edition (BIS-11)

We used the Chinese version of BIS-11. The Chinese version of BIS-11 Scale were revised by Zhou et al. (25). This instrument rates on a 4-point scale three dimensions: attentional impulsivity (AI), motor impulsivity (MI), and non-planning impulsivity (NI). These can measure attention, cognitive stability, motor impulse, perseverance, continence, and cognitive complexity. The total Cronbach's α coefficient for the scales was 0.76, with each Cronbach's α coefficient of the AI, MI, NI being 0.56, 0.66, and 0.69, respectively.

#### Stroop Color-Word Task

Participants performed six runs of the Stroop color-word interference task (26) by the Eprime 2.0 program, in which participants reported the ink color of congruent or incongruent color-word pairs [e.g., “red” written in red (congruent) or blue (incongruent) ink]. Subjects were asked to press the digital corresponding key as soon as possible to the color stimulus (red = 1, green = 2, blue = 3). Runs consisted of 105 stimuli, presented for 1,300 ms, with an inter-trial interval of 350 ms, including 6 incongruent events that were presented pseudo-randomly every 13–16 congruent stimuli.

### Image Acquisition

Images were acquired with a Siemens Trio 3T magnetic resonance imaging (MRI) system (Siemens, Erlangen, Germany) using a 32-channel phased-array head coil. Special care was taken to minimize head motion with the use of an earplug and head cushion that also allowed subjects to maintain a comfortable position during scanning. T1-weighted localized images were scanned by vertical lines of the anterior commissure and posterior commissure lines, and then the structural and functional images were scanned in the same position. High-resolution T1-weighted anatomical images were used for the structural image, repetition time/echo time [TR/TE] 1,500/2.83 ms, flip angle 7°, field of view [FOV] 25.6 × 25.6 cm, 256 × 256 matrix, 176 slices. Functional images were collected using an echo-planar image gradient-echo pulse sequence (TR/TE 1,650/27 ms, flip angle 60°, FOV 22 × 22 cm, 64 × 64 matrix, the total volume = 105, the total scanning time = 173.25 s, 5 mm effective slice thickness with 1 mm gap, 25 slices).

### Statistical Analysis

#### Psychological and Behavioral Data

Relevant parameters were recorded and analyzed by using the Statistical Package for the Social Sciences 21.0 (SPSS 21.0). To understand the differences between the different mean values, an independent-samples *t*-test was used for comparison between groups. Further, a paired sample *t*-test was used for comparison within group. Pearson correlation analysis was used to investigate the relationship between variables.

#### Functional MRI (fMRI) Data

fMRI data were preprocessed and analyzed using SPM12 (Statistical Parametric Mapping, https://www.fil.ion.ucl.ac.uk/spm/software/spm12/) and DPABI (Data Processing & Analysis of Brain Imaging, http://rfmri.org/dpabi), and SPM12 was used based on MATLAB R2013b (matrix & laboratory, https://www.mathworks.com/) (27, 28).

All of the data were preprocessed by SPM 12. Runs with motion in excess of 2.0 mm displacement and 2 degrees rotation were rejected. Four cases of RGUs and 8 cases of IGD were excluded. A total of 22 individuals with IGD (5 females) and 26 RGUs (7 females) were included in the final fMRI analyses. The first 10 images were excluded to ensure steady-state longitudinal magnetization, and the remaining images were then corrected for temporal differences and head motion. The realigned datasets were normalized to Montreal Neurological Institute (MNI) space. The T1 structural images adopted DAETEL registration, and performs spatial smoothing of Gaussian filtering with a full width and half height of 6 mm. Finally, the image was de-linearly drifted, and the whole brain signal did not return. The filtering range was 0.01~0.1 Hz. A 6-mm full-width-half-maximum Gaussian kernel was used for data smoothing.

The GCA is an appropriate method based on multiple linear regression for investigating whether the past value of one time series can correctly predict the current value of another (29). This method has been applied in previous fMRI studies to reveal causal effects among brain regions. In the current study, multivariate GCA was conducted using the Resting-State fMRI Data Analysis Toolkit (REST, http://www.restfmri.net), according to methods described in previous studies. Signed-path coefficients are employed to reveal the Granger causal effects among key nodes of intrinsic connectivity networks (ICNs) within each hemisphere, which are considered to be normally distributed and can be used in parametric statistical analysis for group-level inference.

According to prior literature, an ROI must be determined. Using multiple research paradigms and impulsivity scales, early behavioral studies (7) have confirmed the lack of impulsivity characteristics and inhibitory control ability of individuals with IGD. Inhibitory control circuits are mainly related to the relevant brain regions of the prefrontal cortex, and the DLPFC, anterior cingulate cortex and ventrolateral prefrontal cortex in an important node of these inhibitory control circuits (22). Abnormal inhibitory control in patients with IGD was associated with changes in the DLPFC. In individuals with IGD, functional connections between the DLPFC and the audio-visual, motor, and other brain regions have been shown to be altered (8). The connections between left DLPFC, ventral medial prefrontal cortex, and parietal cortex were weakened (23). Research showed that ventral prefrontal cortex activity was decreased in addictive behavior (22). In IGD patients, the degree of activation of the anterior cingulate gyrus is positively correlated with the desire for online games (30). Therefore, DLPFC, anterior cingulate cortex and ventrolateral prefrontal cortex was intended to be used as an ROI for effective connection analysis in this study.

Seed selection: the seed was designated as the DLPFC, and Neurosynth (http://www.neurosynth.org/) was used with DLPFC as the keyword. The locations of DLPFC in the meta statistical chart were obtained. The coordinates of peak points on the left were x = −46, y = 38, and z = 30. The coordinates of peak on the right were x = 42, y = 38, and z = 32. After selecting voxels with the highest z-scores within each cluster on the functional map, the final ROI were constructed by drawing spheres with centers as the seed point and a radius of 6 mm. Positive/negative connection values mean the degree to which one brain area affects another brain area. The larger the absolute value, the stronger the effect.

Global differences in VMHC (Symmetric Voxel Homotopic Connection) were examined across the whole brain (31). VMHC is an index proposed by researchers such as Stark and Zuo to measure the synchronization of the two hemispheres, by calculating the strength of the functional connection between a voxel and its counterpart in the opposite hemisphere (32). It reflects the synergy of left and right brain signal activities. The higher the VMHC value, the better the balance of functional activity between the cerebral hemispheres. The lower the VMHC value, the worse the balance of functional activity between the cerebral hemispheres. For each subject, the VMHC was computed as the Pearson correlation coefficient between each voxel's residual time series and that of its symmetrical interhemispheric counterpart. Correlation values were then Fisher z-transformed to improve normality. The resultant values constituted the VMHC and were used for group analyses.

## Results

### Psychological Data Analysis

A total of 22 individuals with IGD (5 females) and 26 RGUs (7 females) participated in our behavior data collection. In the IGD group, mean IAT scores were 59.59 ± 7.58, mean age 14.1 ± 1.8 years old, education years 8.5 ± 1.5 years. In the RGU group, mean IAT scores were 39.05 ± 8.66, average age 13.9 ± 1.5 years old, education years 7.9 ± 1.5 years. There were no statistically significant differences in age, gender, or education level between the two groups (*p* > 0.05).

An independent sample *t*-test was used to compare the psychological data of two groups. The IAT and BIS-11 impulsivity scores of the IGD group on AI, MI, and NI factors were higher than those in the RGU group, and the differences were statistically significant (Table 1).

**Table 1:** Differences between the IGD and RGU groups on the IAT and BIS-11.

**Items**	**IAT**	**BIS-11**	**AI**	**MI**	**NI**
	**M ± SD**	**M ± SD**	**M ± SD**	**M ± SD**	**M ± SD**
IGD (*n* = 22)	59.59 ± 7.58	61.45 ± 7.52	16.18 ± 2.87	20.23 ± 3.37	25.05 ± 3.87
RGU (*n* = 26)	38.42 ± 8.41	51.19 ± 8.44	13.08 ± 2.94	17.46 ± 4.03	20.65 ± 3.94
*t*	9.09^***^	4.41^***^	3.69^**^	2.55^*^	3.88^***^
*p*	<0.001	<0.001	0.001	0.014	<0.001

* *p < 0.05*,

** *p < 0.01*,

*** *p < 0.001*.

### Correlations Between IAT and BIS-11

IAT scores were positively correlated with BIS-11 total scores on the AI and MI factors (Table 2).

**Table 2:** Correlation analysis of IAT and BIS-11.

**Items**	**IAT**	**BIS-11**	**AI**	**MI**	**NI**
IAT	1				
BIS-11	0.42^**^	1			
AI	0.44^**^	0.82^**^	1		
MI	0.25	0.79^**^	0.52^**^	1	
NI	0.36^*^	0.82^**^	0.54^**^	0.40	1

* *p < 0.05*,

** *p < 0.01*,

*** *p < 0.001*.

### Stroop Task Differences Between the IGD and RGU Groups

Compared with the RGU group, the IGD group demonstrated longer reaction times (RTs) on congruent (CC) stimuli. At the same time, the IGD group had longer RT and lower accuracy on incongruent (IC) stimuli. The accuracy of the Stroop effect (IC-CC) exhibited a statistically significant difference between the two groups. However, the RT of the Stroop effect (IC-CC) demonstrated no difference between the two groups (Table 3).

**Table 3:** Differences between the IGD and RGU groups in the Stroop task.

**Items**	**Condition**	**IGD (*n* = 22)**	**RGU (*n* = 26)**	***t***	***p***
		**M ± SD**	**M ± SD**		
RT (ms)	CC	679.0 ± 105.4	558.1 ± 118.3	3.71^**^	0.001
	IC	850.6 ± 125.0	740.3 ± 129.6	2.98^**^	0.005
	Stroop	171.6 ± 73.6	182.2 ± 81.6	−0.47	0.640
Accuracy (%)	CC	96.1 ± 2.0	97.0 ± 1.8	−1.64	0.107
	IC	54.4 ± 21.3	75.0 ± 15.7	−3.85^***^	<0.001
	Stroop	−41.7± 20.6	−22.0 ± 15.6	−3.68^***^	<0.001

* *p < 0.05*,

** *p < 0.01*,

*** *p < 0.001*.

Overall comparison found that both groups exhibited a Stroop effect. It can be seen that the accuracy of IC stimulus was lower than CC stimulus (*t*_IGD_ = 10.93, *t*_RGU_ = 11.38, *p* < 0.05) and IC stimulus had longer RT (*t*_IGD_ = −9.47, *t*_RGU_ = −7.17, *p* < 0.05; Table 3).

### GCA Differences Between the IGD and RGU Groups

**Figure 1:**
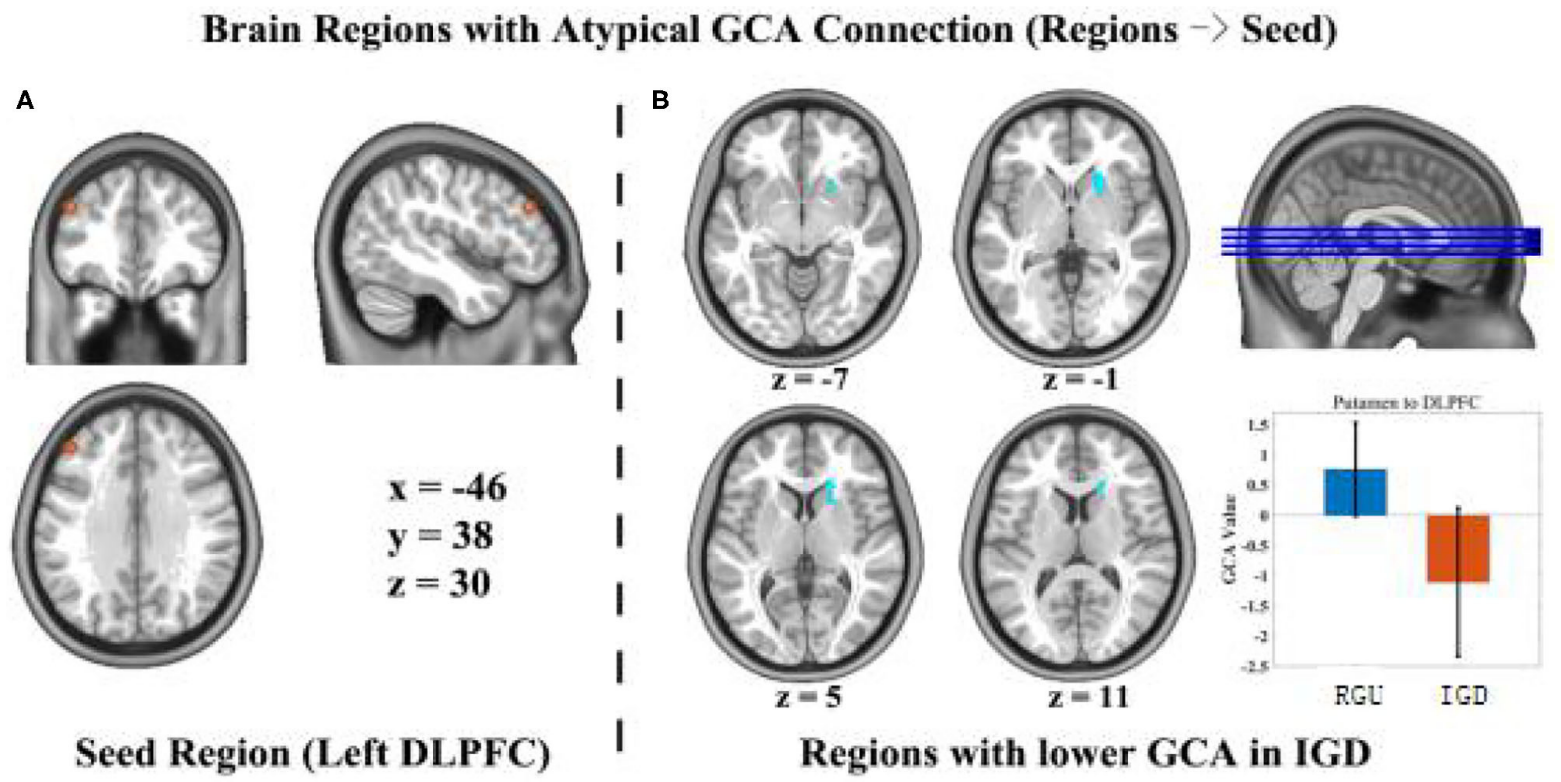
Differences in GCA results between the IGD group and RGU group during resting state. *p* < 0.05 (AlphaSim correction). Voxel size = 3 × 3 × 3 mm^3^, the orange circle is the seed point of the DLPFC left-brain position **(A)**. The blue circle represents an efficient connection with the seed point to abnormal brain areas **(B)**.

In this study, there is no significant difference in anterior cingulate cortex and ventrolateral prefrontal cortex area. We compared connection efficiency between the two groups of the DLPFC and the brain regions related to the prefrontal-striatal circuits. Compared with the RGU group, the dorsal striatum (caudate, putamen) exhibited significant differences in the directed junction values of the left DLPFC (the coordinate of peak, x = 18, y = 27, z = 6, *t* = −5.1928, voxel-level *p* < 0.001, cluster level *p* < 0.05, cluster size = 52). In the RGU group, the dorsal striatum had a positive connection value to the left DLPFC, a one-way positive effect. The activity of the dorsal striatum stimulates the DLPFC. In the IGD group, the connection value was a negative, one-way effect, with dorsal striatum activity inhibiting the left DLPFC (Figure 1).

### VMHC Between the IGD and RGU Groups

**Figure 2:**
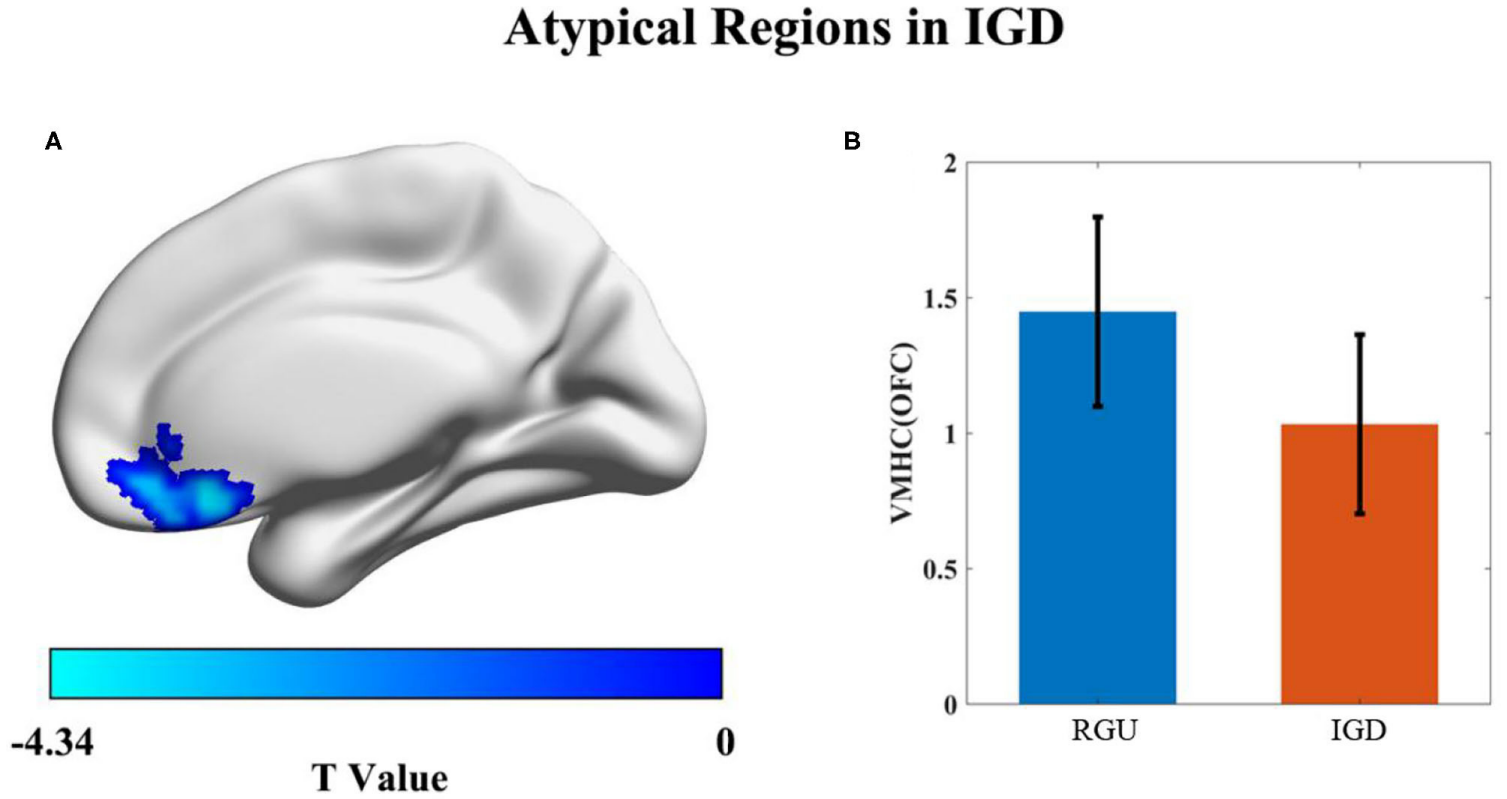
Differences in VMHC value between the IGD and RGU groups durinresting state. *p* < 0.05 (AlphaSim correction). Voxel size = 3 × 3 × 3 mm^3^, the blue color represents the VMHC decreased area in the IGD group compared with the RGU group **(A)**. Histogram **(B)** shows the mean and standard deviation of VMHC values in the two groups.

Compared with the RGU group, the VMHC value of the IGD group demonstrated a significant reduction. The descending region was located in the medial orbital frontal lobe/gyrus rectus (the coordinate of peak, x = −6, y = 27, z = −15, *t* = −4.34, *p* < 0.001, cluster size = 89; Figure 2).

**Figure 3:**
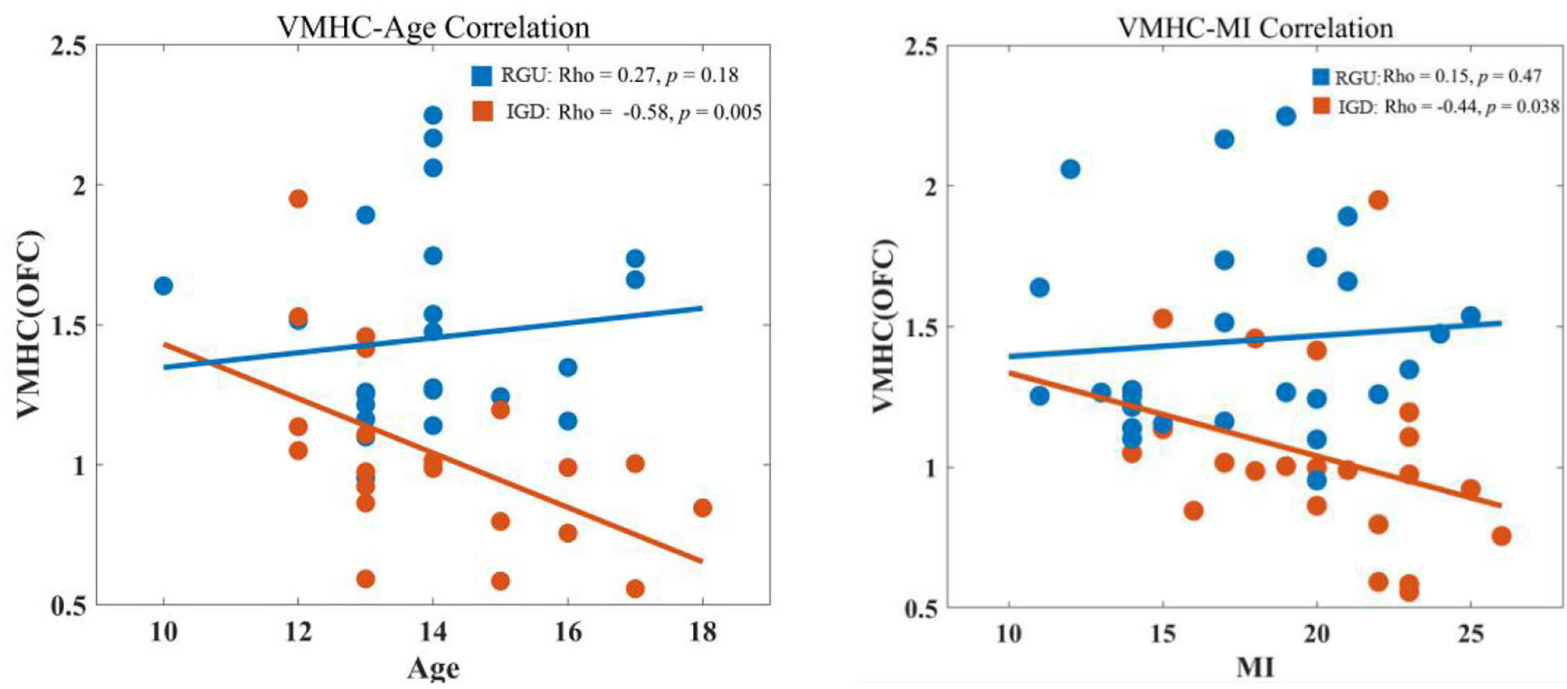
Correlation analysis of VMHC value with age and MI. The left shows the correlation analysis between age and VMHC values of the two groups. The right shows the correlation analysis between the MI and VMHC values of the two groups.

VMHC values in abnormal brain areas of the two groups were extracted, and it was found that VMHC values in the IGD group were negatively correlated with age (*r* = −0.58, *p* < 0.05). The RGU group did not exhibit this tendency (*r* = 0.27, *p* > 0.05). The correlation between the two groups was significantly different. The VMHC value of IGD patients was negatively correlated with MI (*r* = −0.44, *p* < 0.05; Figure 3).

**Figure 4:**
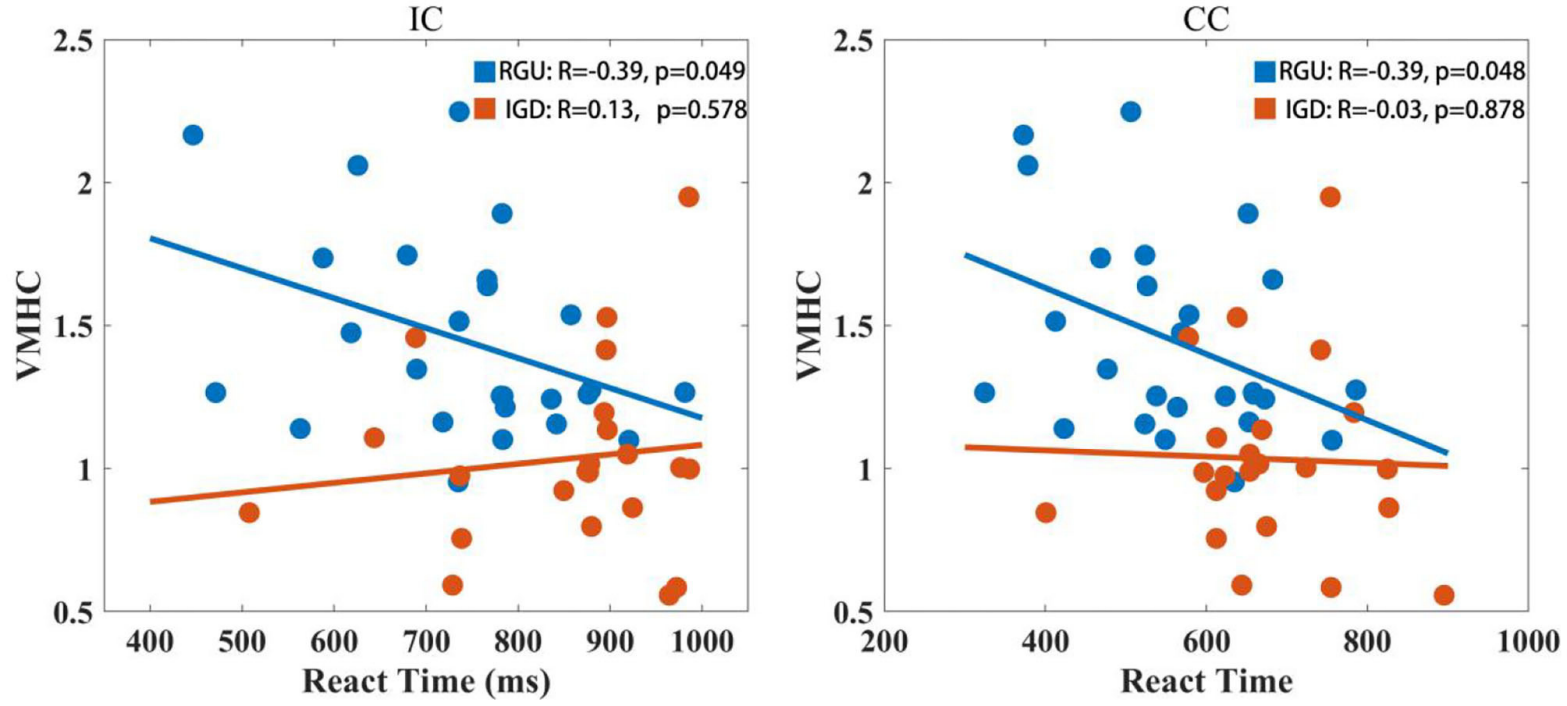
Correlation analysis of VMHC value with RT. The left shows the correlation analysis between RT and VMHC values of the two groups in the IC condition. The right shows the correlation analysis between RT and VMHC values of the two groups in the CC condition.

VMHC values in abnormal brain areas of the two groups were extracted, and it was found that VMHC values in the RGU group were negatively correlated with RT on IC conditions (*r* = −0.39, *p* < 0.05). The IGD group did not have this tendency (*r* = 0.13, *p* > 0.05). Under CC condition, VMHC value in RGU group was negatively correlated with RT (*r* = −0.39, *p* < 0.05). The IGD group did not have this tendency (*r* = −0.03, *p* > 0.05; Figure 4).

## Discussion

### Analysis of Psychological Data Results Between the IGD and RGU Groups

In this study, the impulsivity scores of subjects in the IGD group were significantly higher than those in the RGU group, and the total score of the IAT, as a standard for assessing the severity of IGD, was positively correlated with impulsivity scores, which was essentially consistent with previous studies (33, 34). Impulsivity has often been associated with addiction, which may be a risk factor for IGD (34). High impulsivity scores show that the participant's impulse control is damaged (35). The degree of addiction can also affect the level of impulsivity, which results in individuals with IGD making impulsive decisions more easily and without considering the consequences (36). As such, these individuals are unable to overcome the impulsivity of playing online games. Many phenomena indicate that individuals with IGD, as a behavioral addiction, exhibit poor impulsive control.

### Analysis of Stroop Task Results Between the IGD and RGU Groups

Comparison of Stroop data showed that the IGD group required significantly longer to react than their RGU counterparts under IC and CC conditions, indicating that the IGD group needed more time to distinguish font color when performing a color-word task. This tendency shows that response inhibition is impaired in the IGD group. IGD group, under the stimulus of IC, demonstrated a lower accuracy than the RGU group, which indicates that individuals with IGD face double interference. This illustrates that it is difficult for these individuals to overcome conflict and interference, making it easier to make mistakes, when dealing with complex tasks, such as making decisions in life.

Both groups demonstrated significant Stroop effects, but the comparison between the two groups was not statistically significant. Both groups were more likely to make mistakes and perform worse in more complex and conflicting tasks. These results are consistent with previous results of substance addiction research, such as cocaine addiction (37), alcohol addiction (38), and nicotine addiction (39), which provides behavioral evidence for the similarity between IGD and substance addiction.

### GCA Results Between the IGD and RGU Groups

Compared with the RGU group, there was a significant difference in connection efficacy between the left DLPFC and the dorsal striatum in the IGD group, which indicates that the prefrontal striatum circuits of adolescents with IGD adolescents were partially damaged. It is speculated that the left DLPFC and dorsal striatum play a more important role in the formation of IGD. Previous research has shown an abnormality in the resting state functional connectivity of the frontal lobe-striatal circuit in a population of individuals who abused drugs (18) and individuals with IGD (19, 20), providing a link between the abnormal function of the connection and cognitive control deficits. The experimental results try to fill the blank about the connectivity between the prefrontal cortex and the striatum adolescents with IGD. The high impulse of the nigrostriatal system affects the normal execution of response inhibition function in the prefrontal cortex. Thus, teenagers with IGD repeatedly may engage in online games.

In normally functioning prefrontal-striatal circuits, the DLPFC is successfully activated during the response inhibition process (30, 40). In the IGD group, the connection efficiency value between the dorsal striatum and the left DLPFC was negative, specifically indicating that dorsal striatum activity suppressed the left DLPFC. The striatum has been previously associated with impulsivity and the reward system (41, 42). Playing online games can activate the striatum (43). This means that, due to striatum activation, activity in the left DLPFC is inhibited, which may cause individuals with IGD to be unable to suppress their impulse to play online games (44). This is more likely to lead to impulsive, prolonged gaming behavior. The effective connection of the dorsal striatum and left DLPFC was a positive value. The activity of the dorsal striatum stimulates the DLPFC, which shows that left the DLPFC would be likely to help the individual to inhibit response processing successfully in the RGU group (45).

Electronic game activity increases striatal dopamine release (15). According to substance addiction studies (16, 46), combined with experimental results, we speculate that online games represent a high-level reward stimulus in individuals with IGD. Dopamine could activate the reward pathway of the striatum in the brain, resulting in insufficient regulation of impulsivity, which may lead to prefrontal lobe executive inhibitory control failure (41). This then will lead to more impulsive behaviors. However, this theory still requires more research.

### VMHC Results Between the IGD and RGU Groups

The results of the current study showed that VMHC values in the IGD group were significantly lower than those of the RGU group, which was located in the medial orbitofrontal cortex (mOFC). A previous study (47) compared an IGD group with a healthy control group (the average playing time was <2 h per day). In addition to differences in OFC brain areas, it was found that VMHC values in the middle and superior frontal gyrus were also significantly reduced. However, differences in these brain areas were not observed in this experiment when compared with the RGU group. This indicates that the medial OFC plays a more important role in the IGD process than the superior frontal gyrus and the middle frontal gyrus. The OFC is located in the prefrontal cortex region of the prefrontal striatum circuits, which is involved in the processing of emotion regulation, craving, decision-making, and compulsive behaviors (48). In a study of the VMHC in substance use (32), abnormal OFC synchronization in the left and right hemispheres of the brain was observed in individuals with addiction.

In this experiment, the VMHC value of the mOFC in patients with IGD was significantly negatively correlated with MI, which is an index of motor impulsivity, indicating that adolescent impulsive behavior would affect OFC signal transmission in both hemispheres of the brain. OFC promotes an individual's tendency to goal-oriented behaviors by evaluating the expected results of stimulus and behavior choices. High-impulsivity adolescents with IGD may have impaired OFC assessment function and difficulty in making correct decisions.

In the experiment, the VMHC value of mOFC in the IGD group was negatively correlated with age, while a separate study showed that the duration of heroin use was negatively correlated with the VMHC value of the DLPFC (31). The reason for this may be that older teenagers with IGD may use online games for a longer time, leading to a gradual decline in VMHC value. It may also be that patients with IGD and their parents lack relevant knowledge of IGD, resulting in not seeking timely medical treatment. However, our research group only diagnosed IGD without collecting the disease time of subjects, which will be improved in future studies.

In this study, VMHC values of the mOFC were extracted and correlated with behavioral data obtained in the Stroop color word task. It was found that under the IC and CC conditions, VMHC values of the RGU group were negatively correlated with RT, while those of the IGD group were not correlated. The mOFC is involved in regulating negative emotional states (49). This study speculates that mOFC signal transmission on both sides of the brain might affect emotional processing in the task when RGUs performed the Stroop task, and that the correlation between emotion and response inhibition promoted response inhibition in RGUs. No such link was found in adolescents with IGD, who exhibited lower response suppression. However, the current research (50–52) only stays at the activation level. The role of the functional connection of the mOFC and the mOFC signal synchronization level on both sides of the brain is still unclear in emotional regulation, which needs to be examined by future empirical research.

## Limitations

Owing to technical reasons, our scanning parameters were outdated, making the research results limited. The scanning time of the resting state fMRI used in this study was <3 min. This might affect the reliability of resting-state fMRI connectivity estimates. Additionally, the sample size in the current study was small. We used G-Power to calculate the sample size and initially planned to recruit 77 subjects. As IGD adolescents were not interested in anything than Internet games and also did not think that IGD was a problem for them. So although many adolescents had IGD, but they were not willing to participate in our study. In this study, some teenagers also were reluctant to cooperate with MRI because of their schoolwork/previous commitments. At the same time, part of the collected data was not available because the patient's head motion did not conform to the later image analysis. Therefore, more subjects should be recruited in the future to improve the examination process and further confirm the conclusions of this study. The cause of abnormal effective connection value and VMHC value needs to be explained in combination with more structural imaging results. In these follow-up studies, diffusion tensor imaging data can be collected to further explain abnormal functional connectivity of homologous brain regions in adolescents with IGD. A prior ROI was used for to determine connection efficacy analysis in our study. If an independent component analysis was used to determine brain regions with differences between the two groups, and the ROI was determined this way, more meaningful connections could be found. Finally, this experiment only included IGD and RGU groups. If future research can include a healthy control group that does not play online games, the comparison of the three groups can better explain the pathogenesis of IGD from the biological point of view. In the future, we will recruit more IGD adolescents through schools and the Internet, and combine with diffusion tensor imaging to further explain the abnormal functional connection between the two hemispheres of IGD adolescents. Next, we will also conduct psychological interventions on IGD patients to further confirm the conclusions of this study through functional brain changes before and after the intervention.

## Conclusions

There were significant differences in IAT and impulsivity scores between the IGD and RGU groups, with the IGD group exhibiting higher scores. This indicates that adolescents with IGD have addiction problems and high impulsivity. At the same time, adolescents with IGD demonstrated longer reaction time, lower accuracy, poor response inhibition, and impaired impulse control in the Stroop task. Compared with the RGU group, the efficacy of connections between the left DLPFC and the dorsal striatum in the IGD group were significantly different. It was speculated that online game activities may stimulate the reward pathway in adolescents with IGD at high levels, leading to the failure of normal response inhibition processing and the occurrence of impulsive behaviors. The VMHC value of the mOFC in the IGD group was significantly lower than that in the RGU group, indicating that the mOFC plays an important role in the prefrontal-striatal circuits. Impaired impulsive behavior and response inhibition in adolescents might affect mOFC signal transmission in both hemispheres of the brain.

## Data Availability Statement

The raw data supporting the conclusions of this article will be made available by the authors, without undue reservation.

## Ethics Statement

The studies involving human participants were reviewed and approved by Ethics Committee of The Guizhou Provincial People's Hospital. Written informed consent to participate in this study was provided by the participants' legal guardian/next of kin.

## Author Contributions

HX designed the study and wrote the protocol. JC, YZ, and XL conducted literature searches and provided summaries of previous research studies and conducted the statistical analysis. RW and CT provided technical support. QZ edited and organized the manuscript. All authors contributed to and have approved the final manuscript.

## Conflict of Interest

The authors declare that the research was conducted in the absence of any commercial or financial relationships that could be construed as a potential conflict of interest.
